# Development and validation of a risk prediction model for dry eye disease in myopic children

**DOI:** 10.3389/fmed.2026.1768592

**Published:** 2026-04-28

**Authors:** Bingqing Li, Jie Wen, Jie Qin, Ran Gao, Hanruo Liu, Fengju Zhang

**Affiliations:** 1 Beijing Tongren Eye Center, Beijing Tongren Hospital, Capital Medical University, Beijing Ophthalmology and Visual Sciences Key Laboratory, Beijing, China; 2 Department of Refraction, Shijiazhuang Aier Eye Hospital, Shijiazhuang, China; 3 Department of Refraction, Beijing Aier-Intech Eye Hospital, Beijing, China

**Keywords:** blink, body mass index, child, dry eye disease, myopia, orthokeratologic procedures, predictive value of tests, risk factors

## Abstract

**Introduction:**

To identify independent risk factors for dry eye disease (DED) and to develop and validate a predictive model for DED among myopic schoolchildren aged 8–16 years in northern China.

**Methods:**

A cross-sectional study was conducted among myopic children in Zhangjiakou, Hebei Province. The children underwent comprehensive ocular surface evaluations, including corneal fluorescein staining, tear film break-up time (FBUT), Schirmer I test, lipid layer thickness (LLT), and partial blink rate (PBR). DED was diagnosed using the 2022 Chinese Expert Consensus criteria. Behavioral and environmental risk factors were assessed via validated questionnaires. Logistic regression identified independent factors, and a nomogram was constructed and validated for individualized DED risk estimation.

**Results:**

A total of 1,303 myopic children were included for analysis, and the prevalence of objectively diagnosed DED was 31.2%. Tear film instability, reduced LLT, and increased PBR were the predominant ocular surface abnormalities. The children were divided into training and validation sets according to the community. Among the 912 children in the training set, multivariate analysis identified orthokeratology (Ortho-K) lens use (OR = 4.74), daily screen time ≥ 4 h (OR = 4.21), near work ≥ 4 h (OR = 3.53), BMI ≥ 24 (OR = 3.20), and sleep duration < 6 h (OR = 2.26) as independent risk factors (all *p* < 0.05). The risk prediction nomogram demonstrated acceptable discriminative ability (AUC: 0.74 in the training set and 0.70 in the validation set).

**Conclusion:**

Dry eye disease is common and under-recognized among myopic children in northern China, with risk closely linked to modifiable behavioral and lifestyle factors and Ortho-K lens use. The developed nomogram can facilitate early identification and targeted interventions for high-risk children.

## Introduction

Myopia is a major public health challenge, particularly among children and adolescents in East Asia (1–3). Beyond its impact on vision, emerging evidence shows myopic children are increasingly susceptible to ocular surface disorders, especially dry eye disease (DED), due to behavioral and environmental factors such as increased near work, excessive digital device use, and reduced outdoor activity (4, 5). These factors may destabilize the tear film and compromise ocular surface health. In addition, recent pediatric studies have suggested that myopic children may have worse tear film stability and a higher burden of meibomian gland dysfunction, which may contribute to DED (6–8).

Concurrently, DED, a multifactorial disorder of tear film instability, is an underappreciated pediatric health concern (4), particularly for children with myopia who are at increased risk (6). In addition to lifestyle factors, the growing use of orthokeratology (Ortho-K) lenses further elevates this risk (9). Environmental influences in northern China, including high altitude, low humidity, and air pollution may also exacerbate ocular surface dysfunction (10–12).

However, the independent risk factors for DED in myopic children remain incompletely understood, and practical tools for individualized risk assessment are lacking. While some studies have explored factors associated with DED, with some reporting higher prevalence rates in high-altitude or polluted environments or among those with intensive digital device use or Ortho-K lens wear (5, 9, 11–13), they often have key limitations: many focus on prevalence rather than comprehensive risk factor analysis, lack robust predictive modeling, or are limited by small sample sizes. Moreover, predictive models for DED among myopic children in high-risk geographic regions are scarce. Given these gaps, our study sought to systematically identify risk factors and to construct and validate a predictive model for DED among myopic schoolchildren aged 8–16 years in Hebei, China.

## Materials and methods

### Study design and ethics

This cross-sectional study was conducted between 2023 and 2024 in Zhangjiakou, Hebei Province, China. Zhangjiakou is located at an average altitude of approximately 800–900 m above sea level and is characterized by a continental climate with relatively low humidity, frequent strong winds, and moderate economic development. These environmental and socioeconomic features may influence ocular surface health, particularly among children.

The study adhered to the tenets of the Declaration of Helsinki and received approval from the institutional ethics committee (IRB064). Written informed consent was obtained from the parents or legal guardians of all participants before enrollment.

### Inclusion and exclusion criteria

The inclusion criteria for the study were as follows: children aged 8–16 years with clinically diagnosed myopia, the ability to communicate effectively, and the capacity to complete study procedures. Myopia was classified according to the 2019 Guidelines for Myopia Prevention and Control in Children and Adolescents by the National Health Commission of China: mild myopia (−3.00 D ≤ SE < −0.50 D), moderate myopia (−6.00 D ≤ SE < −3.00 D), and high myopia (SE < −6.00 D). The exclusion criteria included the presence of systemic developmental disorders, psychiatric disorders, hearing impairments, or other cognitive impairments that could affect communication and compliance with study procedures.

### Sample size calculation

The required sample size was estimated using the random sampling formula: n = Z^2^ × P × (1−P)/B^2^. Given the absence of large-scale epidemiological studies on pediatric DED in China, we referenced prior data from Cangzhou City, Hebei Province, reporting a prevalence of 27.24% (*P* = 0.2724) among children (10). With a 20% margin of error (*B* = 0.2724 × 0.20 = 0.05448) and a 95% confidence interval (*Z* = 1.96), the minimum sample size was calculated as 257. After applying a design effect of 4.0 to account for cluster sampling, the adjusted sample size was 1,028. Further considering a 90% expected completion rate, the final required sample size was 1,142. The actual number of participants enrolled (*n* = 1,389) exceeded these requirements.

### Questionnaire survey

Two structured questionnaires were administered in the following order.

(1) Ocular Surface Disease Index (OSDI). The OSDI is a validated 12-item scale used to assess dry eye symptoms and their impact on daily activities. Scores were classified as normal (0–12), mild (13–22), moderate (23–32), or severe (33–100) (14, 15). Previous validation studies have reported good internal consistency and test-retest reliability for the OSDI, with Cronbach’s α values typically above 0.80 and test-retest correlation coefficients above 0.70 (15). A separate reliability analysis for the OSDI was not performed in the present study.

(2) The self-designed Risk Factors Questionnaire was specifically developed for this study to collect information on potential behavioral and environmental risk factors for pediatric DED. Item selection was informed by previous literature on dry eye and ocular surface risk factors in children and young people, including studies on digital device use, near work, sleep, outdoor activity, and environmental exposures (4, 16–18). The questionnaire collected data on demographics, parental smoking, video terminal (screen) use, near work duration, history of ocular and systemic disease, medication use, spectacle or contact lens use (including orthokeratology), dietary habits, and environmental exposures.

### Ophthalmic examinations

All ophthalmologic evaluations were performed by a single experienced ophthalmologist under standardized laboratory conditions. To ensure the accuracy and reliability of measurements, examinations were conducted in a strict sequence from non-invasive to invasive procedures, thereby minimizing the potential impact of earlier tests on subsequent results and preserving tear film integrity throughout. Prior to examination, participants were instructed to remove contact lenses at least 4 h in advance. They were also advised to avoid oil-based eye drops for at least 12 h, ointments for 24 h, swimming for 12 h, and the use of oil-based cosmetics on the day of assessment.

The examination protocol began with the administration of the Ocular Surface Disease Index (OSDI) and the structured risk factor questionnaire. Following this, tear film lipid layer thickness (LLT) and blink dynamics, including partial blink rate (PBR), were quantitatively assessed using the LipiView interferometer. This non-invasive device employs white light interferometry to capture high-resolution interference patterns on the tear film surface, enabling automated calculation of LLT and monitoring of blinking behavior during a standardized 20-s observation period. Incomplete blinks were identified via video analysis, and the partial blink rate was calculated as the ratio of incomplete to total blinks. The maximum and minimum LLT values observed during the session were also recorded (16, 19).

Subsequent to the LipiView assessment, a comprehensive anterior segment evaluation was performed using a slit-lamp biomicroscope. The eyelids were examined for positional abnormalities, trichiasis, and signs of inflammation such as hyperemia, scaling, or ulceration. In cases where scaling was observed, lash microscopy was carried out to detect Demodex mite infestation. The conjunctiva was evaluated for features including congestion, edema, papillary or follicular reactions, scarring, or discharge. Corneal examination focused on identifying congenital anomalies and signs of inflammation (20).

After these non-invasive assessments, invasive ocular surface tests were conducted. Tear film break-up time (FBUT) was measured using the fluorescein sodium staining method. A fluorescein strip, moistened with sterile saline, was gently applied to the bulbar conjunctiva. Under cobalt blue light, the time interval from the last complete blink to the appearance of the first dark spot on the corneal surface was recorded using a stopwatch. Each eye was measured three times, with the mean value used in the analysis. All FBUT measurements were performed by the same examiner in a dark room to minimize variability (21). Immediately following FBUT, corneal fluorescein staining was evaluated. Staining patterns were assessed using a 12-point grading system, in which the cornea was divided into four quadrants, each scored from 0 to 3: 0 indicating no staining, 1 for 1–30 punctate spots, 2 for over 30 non-coalescent spots, and 3 for coalescent staining, filamentary keratitis, or epithelial defects (22). Finally, the Schirmer I test (SIt) was performed without topical anesthesia to assess basal and reflex tear secretion. A standardized test strip was placed in the lateral third of the lower conjunctival sac, and after 5 min, the length of the moistened area was measured in millimeters.

### Handling of missing data and examiner blinding

Participants with incomplete questionnaire responses or missing ocular examination data were excluded from the final analysis to ensure data integrity. Importantly, all ocular surface examinations were performed by examiners who were blinded to the participants’ questionnaire responses, thereby minimizing the risk of observer bias.

### Diagnostic criteria for dry eye disease

Dry eye disease was diagnosed in accordance with the 2022 Chinese Expert Consensus on Dry Eye (23): the primary (objective) criteria required the presence of dry eye symptoms (OSDI score ≥ 13) combined with either a FBUT of ≤5 s or a Schirmer I test result of ≤5 mm/5 min. Alternatively, cases with an FBUT of 5–10 s or a Schirmer I test result of 5–10 mm/5 min were diagnosed as DED if positive corneal fluorescein staining (≥5 punctate spots) was present.

### Covariates and risk factor evaluation

A comprehensive set of demographic, behavioral, and clinical variables was systematically collected and analyzed as potential risk factors for DED in the study population. Demographic data included age, gender, ethnicity, and body mass index (BMI). Clinical factors comprised the severity of myopia (categorized as mild, moderate, or high) and history of ocular disease. Behavioral and environmental factors were assessed through structured questionnaires and included daily screen time (<4 vs. ≥4 h), near work duration (<4 vs. ≥4 h per day), sleep duration (<6 vs. ≥6 h per night), outdoor activity time (<4 vs. ≥4 h per day), spectacle or Ortho-K lens use, dietary balance, and exposure to parental smoking. The thresholds used here are pragmatic rather than diagnostic.

For screen time and near work, the 4-h threshold referred to the cumulative duration per day, including both school-related and leisure activities. For screen time, the cutoff was chosen based on previous studies (17, 24). For near work, however, the cutoff was borrowed from myopia literature (25). The 6-h cutoff for sleep duration was selected to reflect the specific educational and social context of Chinese primary and secondary school students, who frequently experience substantial academic pressure, heavy homework loads, and after-school tutoring, resulting in chronically reduced sleep compared with international recommendations (26, 27). Although 8–10 h of sleep is generally recommended for children aged 8–16 years, many Chinese students do not achieve this duration in practice; therefore, we used 6 h as a pragmatic threshold to identify those with particularly short sleep. For outdoor activity, we initially explored a threshold of 2 h per day, consistent with current myopia prevention guidelines (28). However, this cutoff did not show a statistically significant association with DED in our data. A 4-h threshold was subsequently applied to examine potential dose–response patterns of high outdoor exposure; this variable was not retained in the final multivariate model, and the overall conclusions of the study were not affected by the choice of cutoff.

All variables were selected based on their potential relevance to pediatric DED and were included in subsequent univariate and multivariate analyses to identify independent risk factors.

### Statistical analysis

All statistical analyses were conducted using SPSS software (version 26.0; IBM Corp., Armonk, NY, USA) and GraphPad Prism (version 10.0; GraphPad Software, San Diego, CA, USA). Descriptive statistics were calculated for all variables, with categorical data presented as frequencies and percentages, and continuous data as mean ± standard deviation. Comparisons between groups were performed using independent-samples *t*-tests or one-way analysis of variance (ANOVA) for continuous variables, and chi-square (χ^2^) tests for categorical variables. The relationship between refractive error and dry eye symptoms was examined using Spearman’s rank correlation analysis.

To identify potential risk factors for DED, univariate logistic regression analyses were conducted. Variables showing significant associations in univariate analysis were subsequently included in a multivariate logistic regression model with a stepwise selection method to determine factors independently associated with DED. The dependent variable was the presence or absence of objectively diagnosed dry eye disease. Odds ratios (ORs) with 95% confidence intervals (CIs) and corresponding *p*-values were reported for all analyses, with a two-tailed *p*-value of less than 0.05 considered statistically significant.

For predictive modeling, the study population was divided into training and validation sets. The training set and validation set were collected from different communities within Zhangjiakou, Hebei Province, to ensure external validity. The training set was used for model development, while the validation set served to independently evaluate model performance. A predictive nomogram was constructed based on the independent risk factors identified in the training set. Model performance was assessed using the area under the receiver operating characteristic (ROC) curve (AUC) to evaluate discrimination, calibration plots to compare predicted and observed outcomes, and decision curve analysis (DCA) to assess clinical utility in both cohorts.

## Results

### Baseline characteristics and prevalence of dry eye disease

A total of 1,389 children aged 8–16 years from Zhangjiakou City, Hebei Province, were enrolled, and all participants completed the dry eye symptom questionnaire. Of these, 1,303 children (631 boys and 672 girls; mean age: 11.93 ± 2.61 years) underwent full ophthalmic examinations, including FBUT, Schirmer I test, corneal fluorescein staining, LLT, and PBR assessment. The overall completion rate was 93.81% (boys: 93.07%, girls: 94.51%). Supplementary Table 1 shows that there were no significant differences between those who completed and those who did not complete the examinations.

Using the 2022 Chinese Expert Consensus objective diagnostic criteria, the prevalence of DED in myopic children was 31.2% (406/1,303). In contrast, the prevalence using the subjective OSDI threshold (≥13) was 24.5% (319/1,303), highlighting the underestimation of DED when relying solely on symptoms.

Among the 406 children diagnosed with DED by objective criteria, 68.5% had FBUT ≤ 5 s, 17.0% had FBUT between 5 and 10 s, and only 14.5% had FBUT > 10 s. The Schirmer I test revealed that 4.9% had tear secretion ≤5 mm/5 min, 32.5% had 5–10 mm/5 min, and 62.6% had >10 mm/5 min. Positive corneal fluorescein staining was observed in 5.2% of DED cases. Lipid layer thickness (LLT) was ≤60 nm in 56.2% and >60 nm in 43.8%, indicating a high prevalence of thin tear film lipid layers. Regarding blink behavior, 66.3% of myopic children with DED had a partial blink rate (PBR) ≥ 40%. The most frequently reported symptom was sticky eyelids upon waking (56.9%), followed by dryness (45.1%), redness (41.4%), burning sensation (30.5%), grittiness (16.8%), and crusting of eyelashes (12.3%) (Supplementary Figure 1).

### Univariate analysis of risk factors

Participants were divided into training and validation sets, each recruited from different communities within Zhangjiakou to enhance external validity (Supplementary Table 2 summarizes the baseline characteristics of the two sets). Notably, there were significant differences between the training and validation sets in age (mean age: 11.57 ± 2.58 vs. 10.66 ± 1.92 years; *p* < 0.001), BMI distribution (*p* = 0.002), and sleep duration (*p* < 0.001). Other variables did not differ significantly between the two sets (all *p* > 0.05).

These variations reflect the demographic and lifestyle differences across communities and reinforce the importance of validating the predictive model in distinct populations. No significant differences were observed in key ocular or behavioral variables, supporting the comparability and generalizability of the study findings.

Further analysis was conducted among 912 children in the training set. Baseline characteristics and the distribution of potential risk factors between children with and without objectively diagnosed dry eye disease (DED) are presented in Table 1, while overall comparability between the training and validation sets is shown in Supplementary Table 2. The comparisons showed significantly higher prevalence of DED among children with BMI ≥ 24, those using orthokeratology (Ortho-K) lenses, daily screen time ≥ 4 h, near work time ≥ 4 h, and sleep duration < 6 h (all *p* < 0.05). No significant associations were observed with age, gender, ethnicity, myopia severity, spectacle wear, outdoor activity, unbalanced diet, parental smoking, or ocular disease history.

**TABLE 1:** Comparison of demographic and risk factors between non-DED and DED groups.

Characteristics	NON-DED	DED	X^2^/t	*P*
Age	10.60 ± 1.79	10.79 ± 2.18	−1.230	0.219
Gender	–	–	1.185	0.276
Male	294 (67.1%)	144 (32.9%)		
Female	334 (70.5%)	140 (29.5%)		
BMI			5.105	0.024
<24	620 (69.4%)	274 (30.6%)		
≥24	8 (44.4%)	10 (55.6%)		
Ethnicity			0.900	0.343
Han	578 (69.3%)	256 (30.7%)		
Minority	50 (64.1%)	28 (35.9%)		
Myopia severity	–	–	1.115	0.573
Mild	339 (69.9%)	146 (30.1%)		
Moderate	247 (68.4%)	114 (31.6%)		
High	42 (63.6%)	24 (36.4%)		
Ortho-K			59.061	<0.001
N	590 (73.1%)	217 (26.9%)		
Y	38 (36.2%)	67 (63.8%)		
Glasses wear			1.808	0.179
N	99 (64.3%)	55 (35.7%)		
Y	529 (69.8%)	229 (30.2%)		
Daily screen time			75.750	<0.001
<4	421 (80.3%)	103 (19.7%)		
≥4	207 (53.4%)	181 (46.6%)		
Near work time			52.893	<0.001
<4	570 (73.5%)	205 (26.5%)		
≥4	58 (42.3%)	79 (57.7%)		
Sleep duration			46.207	<0.001
<6	25 (33.8%)	49 (66.2%)		
≥6	603 (72%)	235 (28%)		
Outdoor time			2.261	0.133
<4	164 (72.9%)	61 (27.1%)		
≥4	464 (67.5%)	223 (32.5%)		
Unbalanced diet			0.032	0.857
N	443 (68.7%)	202 (31.3%)		
Y	185 (69.3%)	82 (30.7%)		
Parental smoking			0.286	0.592
N	474 (68.4%)	219 (31.6%)		
Y	154 (70.3%)	65 (29.7%)		
Ocular disease history			0.007	0.933
N	516 (68.8%)	234 (31.2%)		
Y	112 (69.1%)	50 (30.9%)		

Univariate logistic regression results are presented in Supplementary Table 3, which further confirmed that BMI ≥ 24 (OR = 2.83, 95% CI: 1.10–7.24, *p* = 0.030), Ortho-K lens use (OR = 4.79, 95% CI: 3.13–7.35, *p* < 0.001), daily screen time ≥ 4 h (OR = 3.57, 95% CI: 2.67–4.79, *p* < 0.001), near work time ≥ 4 h (OR = 3.79, 95% CI: 2.60–5.51, *p* < 0.001), and sleep duration < 6 h (OR = 5.03, 95% CI: 3.04–8.33, *p* < 0.001) were significantly associated with increased risk of DED. No significant associations were observed for other variables, including age, gender, myopia severity, outdoor time, diet, parental smoking, or ocular disease history.

### Multivariate analysis and predictive model construction

Multivariate logistic regression identified five independent risk factors for DED: BMI ≥ 24 (OR = 3.196, 95% CI: 1.116–9.150, *p* = 0.030), Ortho-K lens use (OR = 4.744, 95% CI: 2.947–7.639, *p* < 0.001), daily screen time ≥ 4 h (OR = 4.211, 95% CI: 3.037–5.838, *p* < 0.001), near work time ≥ 4 h (OR = 3.529, 95% CI: 2.186–5.697, *p* < 0.001), and sleep duration < 6 h (OR = 2.262, 95% CI: 1.169–4.377, *p* = 0.015) (Table 2, Supplementary Figure 2).

**TABLE 2:** Multivariate logistic regression analysis of risk factors for objective DED.

Characteristics	Categories	B	S.E.	Wald	*P*	OR	95% CI for OR	
							Lower	Upper
BMI	≥24	1.162	0.537	4.688	0.03	3.196	1.116	9.15
	<24	Ref						
Ortho-K	Y	1.557	0.243	41.052	<0.001	4.744	2.947	7.639
	N	Ref						
Daily screen time	≥4	1.438	0.167	74.413	<0.001	4.211	3.037	5.838
	<4	Ref						
Near work time	≥4	1.261	0.244	26.635	<0.001	3.529	2.186	5.697
	<4	Ref						
Sleep duration	<6	0.816	0.337	5.875	0.015	2.262	1.169	4.377
	≥6	Ref						

### Model development and performance

**FIGURE 1:**
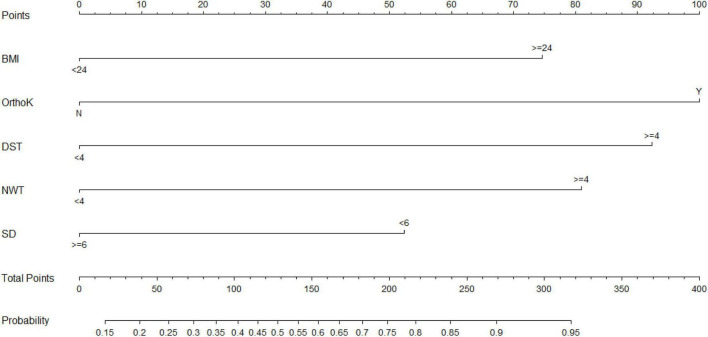
Nomogram for predicting the risk of dry eye disease in myopic children. Each factor (BMI, Ortho-K lens use, daily screen time, near work time, and sleep duration) is assigned a point value according to its level; the total score corresponds to the predicted probability of DED. BMI: body mass index (kg/m^2^), categorized as <24 vs. ≥24 kg/m^2^. Ortho-K: orthokeratology lens use (yes/no). Daily screen time: <4 vs. ≥4 h/day. Near work time: < 4 vs. ≥4 h/day. Sleep duration: ≥6 vs. <6 h/night.

A nomogram was constructed based on the five factors independently associated (Figure 1; scoring details are provided in Supplementary Table 4 and predicted probabilities by total score in Supplementary Table 5). The model showed acceptable discriminative ability in both cohorts: in the training set, the AUC was 0.74; in the validation set, the AUC was 0.70 (Supplementary Figure 3). Calibration plots indicated good agreement between predicted and observed risks (Supplementary Figure 4). Decision curve analysis suggested that the nomogram provided greater net clinical benefit than any single factor across a range of threshold probabilities (Supplementary Figure 5).

## Discussion

This large-scale, school-based study provides robust evidence that DED is highly prevalent among myopic children in northern China, with an objective prevalence of 31.2%, which is notably higher than estimates based solely on subjective symptoms (24.5%). This discrepancy highlights the limitations of symptom-based screening in pediatric populations, where children may underreport or fail to recognize ocular discomfort, and therefore supports the use of objective diagnostic criteria in both clinical practice and epidemiological research.

**FIGURE 2:**
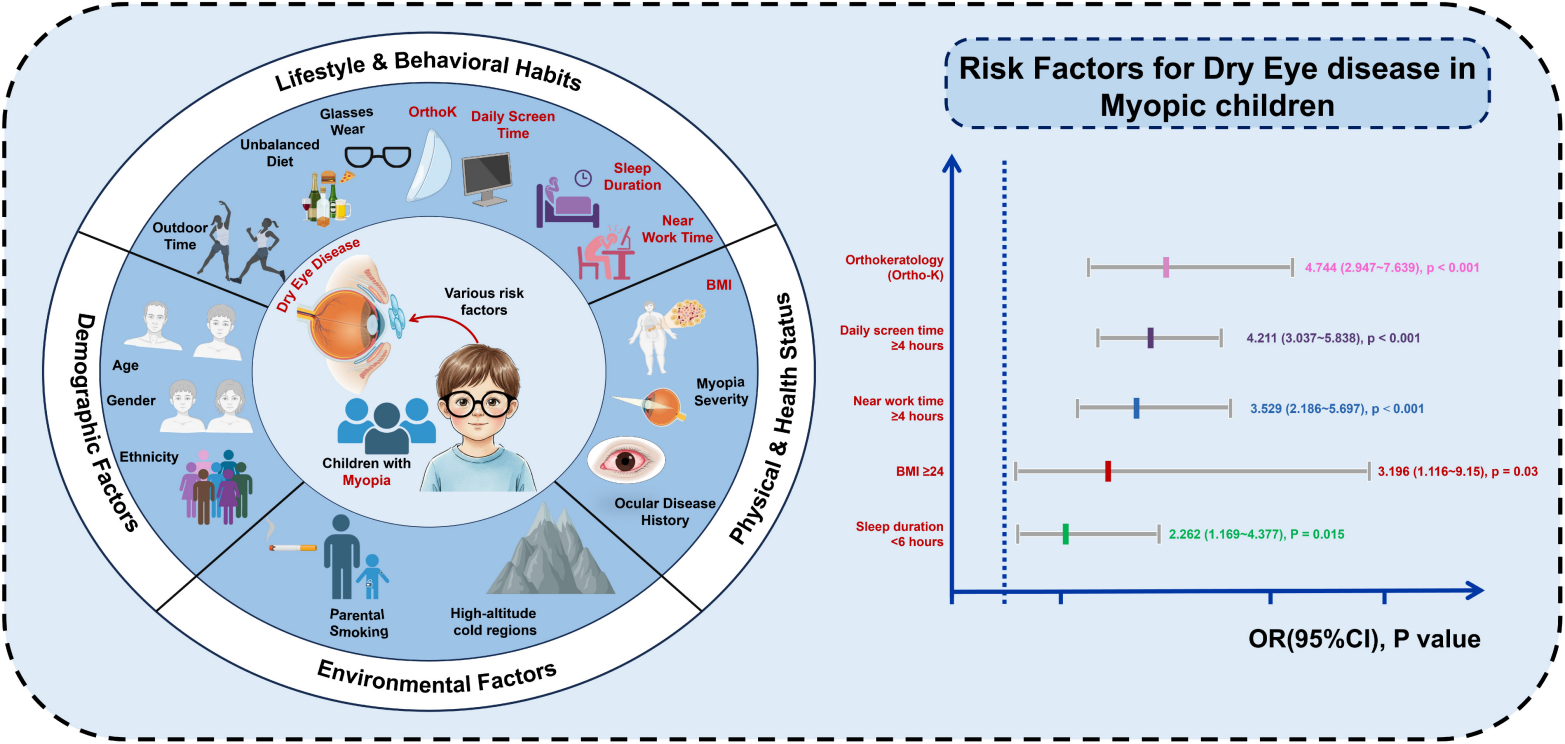
Visualization of risk factors of DED in myopic children. Created in BioRender. Li, B. (2026) https://BioRender.com/lnte70i.

Through comprehensive risk factor analysis, our study identified five key independent factors associated with DED in myopic children (Figure 2): orthokeratology (Ortho-K) lens use, daily screen time ≥ 4 h, near work time ≥ 4 h, BMI ≥ 24, and sleep duration < 6 h. These odds ratios quantify the strength of association rather than proving causality, given the cross-sectional design. Nonetheless, the findings underscore the multifactorial nature of pediatric DED, implicating both lifestyle and clinical management factors. Notably, Ortho-K lens use emerged as a strong risk factor, consistent with previous reports that contact lens wear can compromise tear film stability and increase ocular surface inflammation in children (29). Prolonged screen and near work times likely contribute to reduced blink rates and increased evaporative stress, as seen in both adult and pediatric research (4, 30). A higher BMI and insufficient sleep may contribute to DED via several pathways, including low-grade systemic inflammation, altered meibomian gland function, and dysregulation of hormonal and autonomic control of tear secretion, suggesting that broader health behaviors influence ocular surface homeostasis (31–33). It is important to note that the BMI threshold used in our analysis (BMI ≥ 24) reflects overweight or obese status in this age group, rather than mild weight variation; thus, our results primarily capture the impact of more pronounced adiposity on DED risk. Similarly, the <6-h sleep cutoff represents a relatively severe level of sleep restriction for school-aged children, and our findings should be interpreted as reflecting the risk associated with marked sleep deprivation rather than modest deviations from recommended sleep duration.

A notable strength of our study is the development and validation of a multifactorial nomogram for individualized DED risk assessment in myopic children. The risk model demonstrated acceptable discrimination (AUC: 0.74 in training, 0.70 in validation), calibration, and clinical utility, as assessed by ROC and decision curve analysis. To our knowledge, predictive models for DED in children, particularly among those with myopia, are scarce in the literature. Most DED prediction tools have been developed in adult populations, often focusing on postmenopausal women, office workers, or those with systemic disease (34). For example, Xu et al. constructed a nomogram for DED risk in adults based on demographic and environmental factors, reporting similar predictive accuracy (35). In contrast, pediatric-specific prediction models are limited and usually focus on adolescents in general rather than myopic schoolchildren, and they rarely incorporate ocular management factors such as Ortho-K use or detailed screen/near work behavior (36). Our model is therefore among the first to address the unique risk profile of myopic schoolchildren in a high-prevalence region by integrating both clinical (Ortho-K, BMI) and behavioral (screen time, near work, sleep) variables. In clinical practice, this nomogram can be applied during routine visits by entering these easily obtainable variables to estimate an individual child’s probability of objective DED. Children whose predicted risk exceeds a practical threshold (e.g., 30%–40%) can then be prioritized for detailed ocular surface evaluation, targeted counseling on modifiable behaviors, and closer follow-up in busy optometry or pediatric ophthalmology clinics.

Consistent with prior research, tear film instability emerged as the predominant ocular surface abnormality in myopic children with DED, with over two-thirds displaying a FBUT of 5 s or less (37, 38). This finding highlights the critical role of tear film dynamics in pediatric DED pathogenesis. The high prevalence of incomplete blinking observed in our cohort further supports the notion that blink behavior is a key modifiable factor; reduced and incomplete blinking, often associated with digital device use, exacerbates tear evaporation and destabilizes the tear film, as reported in multiple pediatric studies (39). The unique environmental characteristics of Zhangjiakou, including high altitude, low humidity, and frequent strong winds, likely amplify the evaporative component of DED in our cohort. Such conditions can accelerate tear film evaporation and destabilize the lipid layer, particularly in children with prolonged screen use and near work, who already exhibit reduced blink frequency and increased partial blinks. Although our cross-sectional design precludes quantifying the exact contribution of local climate to DED risk, these findings suggest that environmental dryness and wind exposure may interact synergistically with behavioral factors to increase the burden of evaporative DED in this region. This may partly explain the relatively high prevalence observed in our study compared with reports from more temperate or humid areas. Our detailed ocular surface analysis thus reinforces the need to address both environmental and behavioral contributors to DED in children.

Among all risk factors, Ortho-K lens use was the strongest independent factor, conferring a nearly fivefold increased risk of DED after multivariate adjustment. This is consistent with previous evidence that orthokeratology is effective for myopia control (40) and that Ortho-K wear may be associated with changes in tear film quality and stability in children (9). Potential mechanisms include mechanical interaction between the lens and corneal epithelium, alterations in tear film spreading over the reshaped corneal surface, and lens-related bioburden or subclinical inflammation (9, 41). In addition, wearing time, overnight wear patterns, and lens care habits (e.g., cleaning solutions, compliance with hygiene instructions) are likely to modulate DED risk in Ortho-K users (42). However, our study did not collect detailed data on wearing duration, replacement schedules, or care practices, and we were therefore unable to evaluate these potential interactions. Our findings thus align with and expand upon previous reports, reinforcing the need for careful ocular surface monitoring in children undergoing orthokeratology treatment and for standardized education on lens hygiene and wear schedules.

Digital screen exposure remains an increasingly prominent risk factor for pediatric DED, particularly in the post-pandemic era. Our analysis showed that children with daily screen time ≥ 4 h faced over a fourfold increased risk of objective DED. This is consistent with reports linking excessive electronic device use to increased dry eye symptoms and objective indicators of tear film instability in children (17, 43). Mechanistically, extended screen time suppresses spontaneous blinking, increases partial blinks, and accelerates tear evaporation, as highlighted in both experimental and epidemiological studies (17, 44). These findings underscore the urgent need for digital hygiene education and screen time regulation in pediatric eye health programs, including regular breaks, optimization of viewing distance and lighting, and parental or school-level guidance.

Higher BMI also independently predicted DED risk, corroborating evidence that obesity and overweight status contribute to meibomian gland dysfunction and ocular surface inflammation in children (32). Pediatric studies have demonstrated associations between increased adiposity, meibomian gland dropout, and altered gland morphology, supporting a link between metabolic status and lipid layer integrity (32, 45, 46). Obesity-related systemic inflammation and dyslipidemia may further affect the composition and secretion of meibum, thereby promoting evaporative DED (33, 47). Integrated pediatric eye care should thus incorporate nutritional counseling and weight management alongside traditional ophthalmic strategies. Additionally, insufficient sleep (<6 h per night) was strongly associated with increased DED risk. Experimental and clinical data suggest that sleep deprivation can reduce tear secretion, compromise ocular surface repair, and increase inflammatory mediators, thereby contributing to DED development and progression (48, 49). Moreover, longer sleep duration has been associated with a substantially lower risk of DED in some studies (50–52). Our study extends this evidence by demonstrating a clear association between inadequate sleep and objective DED in myopic children, particularly at the relatively extreme level of <6 h of sleep per night, supporting the inclusion of sleep hygiene counseling in DED prevention strategies.

The relationship between myopia severity and DED remains complex. In our cohort, objective DED prevalence did not differ significantly by myopia grade, whereas subjective symptoms appeared more pronounced among children with high myopia. Some authors have hypothesized that anatomical and neurophysiological changes associated with high myopia may alter ocular surface sensation and tear film dynamics, although direct evidence in children remains limited (53). High myopia is also frequently accompanied by intensive near work and prolonged device use, which may indirectly increase DED risk through behavioral pathways (54). Moreover, the interplay between high myopia, frequent blinking, and meibomian gland dysfunction suggests multifactorial mechanisms (54, 55) and warrants further longitudinal investigation.

Despite these important insights, several limitations merit consideration. First, the cross-sectional design restricts causal inference; longitudinal studies are needed to clarify temporal relationships between risk factors and DED onset. Second, although our sample was large and well characterized, it was geographically restricted to northern China; thus, generalizability to other regions, climates, and populations may be limited. Third, while standardized objective assessments were used, measurement variability related to pediatric cooperation and examiner technique cannot be fully excluded. Fourth, several exposures were categorized using pragmatic thresholds, particularly BMI ≥ 24 kg/m^2^ and sleep duration < 6 h/night, which likely capture more extreme overweight/obesity and marked sleep restriction in this age group; therefore, our estimates may not reflect risk gradients associated with milder variations in BMI or sleep duration. In addition, the small size of the BMI ≥ 24 subgroup may have reduced the precision of the BMI effect estimate (as reflected by a wide confidence interval) and may increase the risk of model instability. Future studies should evaluate these factors using age- and sex-adjusted pediatric BMI metrics (e.g., BMI percentiles/z-scores) and continuous or multi-level sleep measures. Fifth, we lacked detailed data on potentially relevant factors such as Ortho-K wearing schedules and lens care practices, dietary composition and micronutrient status, and indoor air quality, which may have resulted in residual confounding. Finally, other unmeasured environmental or behavioral factors may also have influenced our findings.

In conclusion, this study provides comprehensive evidence that DED is a major and under-recognized health concern among myopic children in northern China. The nomogram we developed could enable quick identification of high-risk children in optometry clinics, facilitating earlier detection, timely intervention, shorter consultations, and targeted management to protect pediatric ocular surface health. By incorporating readily obtainable clinical and behavioral information, the tool may help clinicians stratify risk and allocate chair time more efficiently in routine practice. Key modifiable risk factors should be systematically addressed in pediatric eye care. Targeted behavioral interventions, routine ocular surface monitoring for high-risk children, and broader public health strategies are warranted to mitigate the growing burden of pediatric DED in the digital era. Future longitudinal research should further elucidate the mechanisms linking myopia, lifestyle, and ocular surface health and validate risk prediction models across diverse populations.

## Data Availability

The original contributions presented in this study are included in this article/Supplementary material, further inquiries can be directed to the corresponding author.

## Supplementary material

Supplementary Figure 1 Ocular surface characteristics in myopic children with objective DED: **(A)** FBUT distribution; **(B)** Schirmer I test; **(C)** Corneal staining; **(D)** LLT; **(E)** PBR; **(F)** Symptom distribution.

Supplementary Figure 2 Forest plot of independent risk factors for objective dry eye in myopic children.

Supplementary Figure 3 ROC curves for model discrimination: (left) training set, (right) validation set.

Supplementary Figure 4 Calibration plots for nomogram in (left) training set and (right) validation set.

Supplementary Figure 5 Decision curve analysis (DCA) comparing net clinical benefit of the nomogram and individual factors in the (left) training and (right) validation sets.
