# Supplementary material for: Development and validation of a risk prediction model for dry eye disease in myopic children
Source: Front Med (Lausanne). 2026 Apr 28;13:1768592. doi: 10.3389/fmed.2026.1768592 (PMC13162332; doi:10.3389/fmed.2026.1768592)
Supplement: Supplementary file 1 [file Table_1.docx]

**Supplementary Table S1. Baseline characteristics of children with and without completed ophthalmic examinations**

| Variable | Not Examined | Examined | Test Statistic | P-value |
| --- | --- | --- | --- | --- |
| Gender (M/F) | 47/39 | 631/672 | 1.251 | 0.263 |
| Age (years) | 11.86±2.52 | 11.57±2.58 | 0.995 | 0.320 |
| OSDI Score | 10.12±5.35 | 11.29±6.69 | -1.596 | 0.111 |

**Supplementary Table 2. Baseline characteristics of the training and validation sets.**

| Variable | Training Set | Validation Set | X²/t | *p* |
| --- | --- | --- | --- | --- |
| Age (years) | 11.57±2.58 | 10.66±1.92 | -20.440 | <0.001 |
| Gender |  |  | 0.195 | 0.659 |
| Male | 438 (48.0%) | 193 (49.4%) |  |  |
| Female | 474 (52.0%) | 198 (50.6%) |  |  |
| BMI |  |  | 9.539 | 0.002 |
| <24 | 894 (98.0%) | 371 (94.9%) |  |  |
| ≥24 | 18 (2.0%) | 20 (5.1%) |  |  |
| Ethnicity |  |  | 0.677 | 0.411 |
| Han | 834 (91.4%) | 352 (90.0%) |  |  |
| Minority | 78 (8.6%) | 39 (10.0%) |  |  |
| Myopia Severity |  |  | 4.210 | 0.122 |
| Mild | 485 (53.2%) | 184 (47.1%) |  |  |
| Moderate | 361 (39.6%) | 177 (45.3%) |  |  |
| High | 66 (7.2%) | 30 (7.7%) |  |  |
| Ortho-K use |  |  | 0.608 | 0.435 |
| No | 807 (88.5%) | 340 (87.0%) |  |  |
| Yes | 105 (11.5%) | 51 (13.0%) |  |  |
| Glasses wear |  |  | 0.473 | 0.491 |
| No | 154 (16.9%) | 60 (15.3%) |  |  |
| Yes | 758 (83.1%) | 331 (84.7%) |  |  |
| Daily screen time |  |  | 0.397 | 0.529 |
| <4 h | 524 (57.5%) | 232 (59.3%) |  |  |
| ≥4 h | 388 (42.5%) | 159 (40.7%) |  |  |
| Near work time |  |  | 0.718 | 0.397 |
| <4 h | 775 (85.0%) | 325 (83.1%) |  |  |
| ≥4 h | 137 (15.0%) | 66 (16.9%) |  |  |
| Sleep duration |  |  | 17.087 | <0.001 |
| <6 h | 74 (8.1%) | 8 (2.0%) |  |  |
| ≥6 h | 838 (91.9%) | 383 (98.0%) |  |  |
| Outdoor time |  |  | 0.116 | 0.733 |
| <4 h | 225 (24.7%) | 93 (23.8%) |  |  |
| ≥4 h | 687 (75.3%) | 298 (76.2%) |  |  |
| Unbalanced diet |  |  | 0.774 | 0.379 |
| No | 645 (70.7%) | 267 (68.3%) |  |  |
| Yes | 267 (29.3%) | 124 (31.7%) |  |  |
| Parental smoking |  |  | 0.008 | 0.930 |
| No | 693 (76.0%) | 298 (76.2%) |  |  |
| Yes | 219 (24.0%) | 93 (23.8%) |  |  |
| Ocular disease history |  |  | 1.132 | 0.287 |
| No | 750 (82.2%) | 331 (84.7%) |  |  |
| Yes | 162 (17.8%) | 60 (15.3%) |  |  |

**Supplementary Table S3. Univariate logistic regression analysis of potential DED risk factors**

|  | B | S.E. | Wald | P | OR | 95%CI for OR | |
| --- | --- | --- | --- | --- | --- | --- | --- |
|  |  |  |  |  |  | Lower | Upper |
| Age | 0.049 | 0.037 | 1.749 | 0.186 | 1.05 | 0.977 | 1.13 |
| Gender(M) | 0.156 | 0.143 | 1.184 | 0.277 | 1.169 | 0.883 | 1.547 |
| BMI(>=24) | 1.04 | 0.48 | 4.695 | 0.030 | 2.828 | 1.104 | 7.244 |
| Ethnicity(Minority) | 0.235 | 0.248 | 0.897 | 0.344 | 1.264 | 0.778 | 2.055 |
| MyopiaSeverity |  |  |  |  |  |  |  |
| Moderate | 0.069 | 0.15 | 0.212 | 0.645 | 1.072 | 0.798 | 1.439 |
| High | 0.283 | 0.274 | 1.062 | 0.303 | 1.327 | 0.775 | 2.272 |
| OrthoK | 1.567 | 0.218 | 51.668 | <0.001 | 4.794 | 3.127 | 7.35 |
| GlassesWear | -0.249 | 0.186 | 1.802 | 0.179 | 0.779 | 0.541 | 1.122 |
| DailyScreenTime(>=4) | 1.274 | 0.15 | 72.294 | <0.001 | 3.574 | 2.665 | 4.794 |
| NearWorkTime(>=4) | 1.332 | 0.191 | 48.539 | <0.001 | 3.787 | 2.604 | 5.508 |
| SleepDuration(<6) | 1.615 | 0.258 | 39.34 | <0.001 | 5.029 | 3.036 | 8.331 |
| OutdoorTime(>=4) | 0.256 | 0.171 | 2.255 | 0.133 | 1.292 | 0.925 | 1.805 |
| UnbalancedDiet | -0.028 | 0.158 | 0.032 | 0.857 | 0.972 | 0.714 | 1.324 |
| ParentalSmoking | -0.09 | 0.169 | 0.286 | 0.593 | 0.914 | 0.656 | 1.272 |
| OcularDiseaseHistory | -0.016 | 0.187 | 0.007 | 0.933 | 0.984 | 0.682 | 1.422 |

**Supplementary Table S4. Nomogram factor scores for predicting objective DED**

| Factor | Value | Points |
| --- | --- | --- |
| BMI | ≥24 | 75 |
|  | <24 | 0 |
| OrthoK | Yes | 100 |
|  | No | 0 |
| DailyScreenTime | ≥4 h | 92 |
|  | <4 h | 0 |
| NearWorkTime | ≥4 h | 81 |
|  | <4 h | 0 |
| SleepDuration | <6 h | 52 |
|  | ≥6 h | 0 |

**Supplementary Table S5. Probability of DED by total nomogram score**

| Total Points | Probability |
| --- | --- |
| 17 | 15.0% |
| 39 | 20.0% |
| 58 | 25.0% |
| 74 | 30.0% |
| 88 | 35.0% |
| 102 | 40.0% |
| 115 | 45.0% |
| 128 | 50.0% |
| 141 | 55.0% |
| 154 | 60.0% |
| 168 | 65.0% |
| 183 | 70.0% |
| 199 | 75.0% |
| 217 | 80.0% |
| 240 | 85.0% |
| 269 | 90.0% |
| 317 | 95.0% |
