# Supplementary figures and images for: Development and validation of a risk prediction model for dry eye disease in myopic children
Source: Front Med (Lausanne). 2026 Apr 28;13:1768592. doi: 10.3389/fmed.2026.1768592 (PMC13162332; doi:10.3389/fmed.2026.1768592)

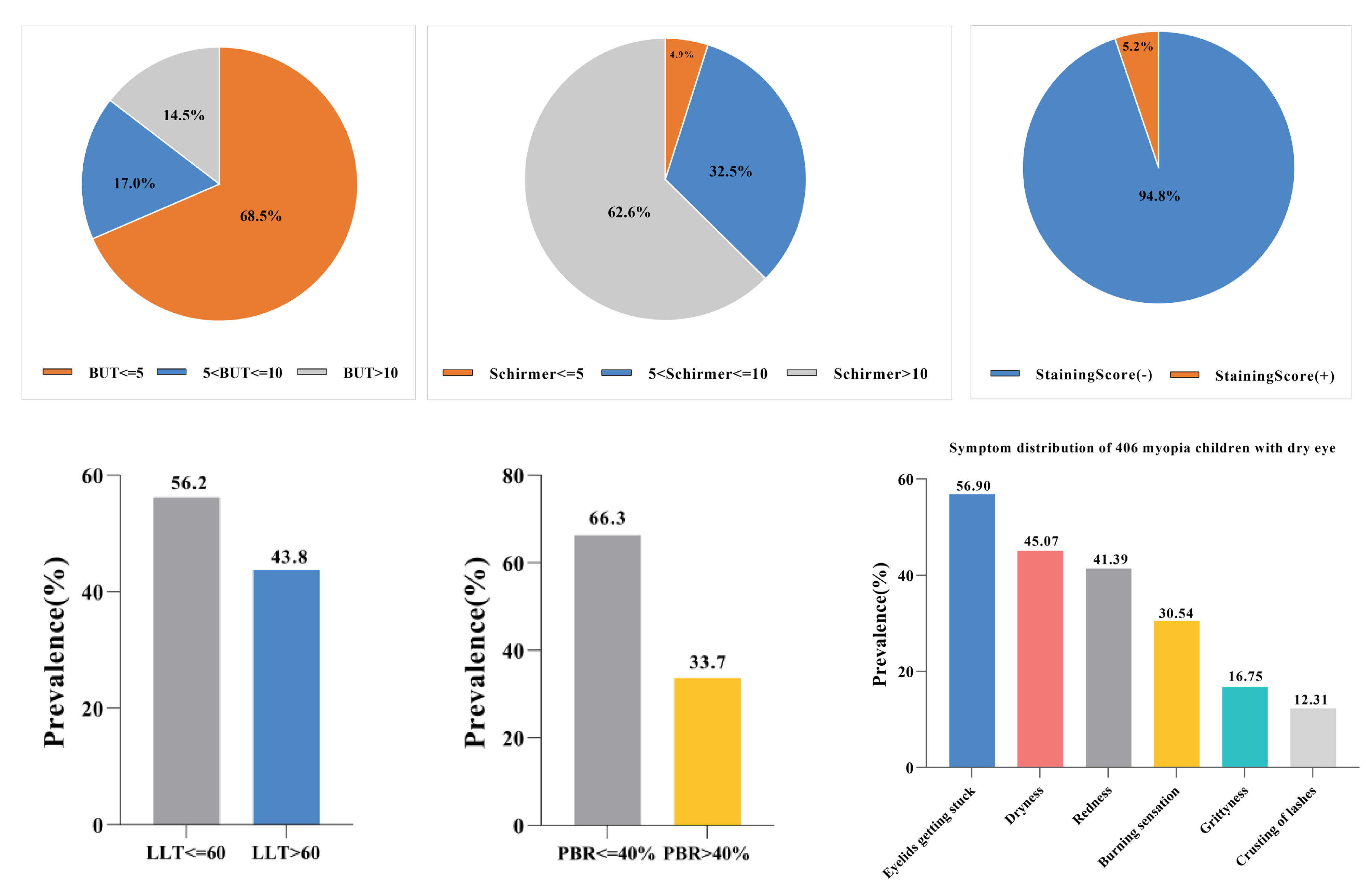

Supplement: Supplementary Figure 1 — Ocular surface characteristics in myopic children with objective DED: (A) FBUT distribution; (B) Schirmer I test; (C) Corneal staining; (D) LLT; (E) PBR; (F) Symptom distribution. [file Image_1.tif]

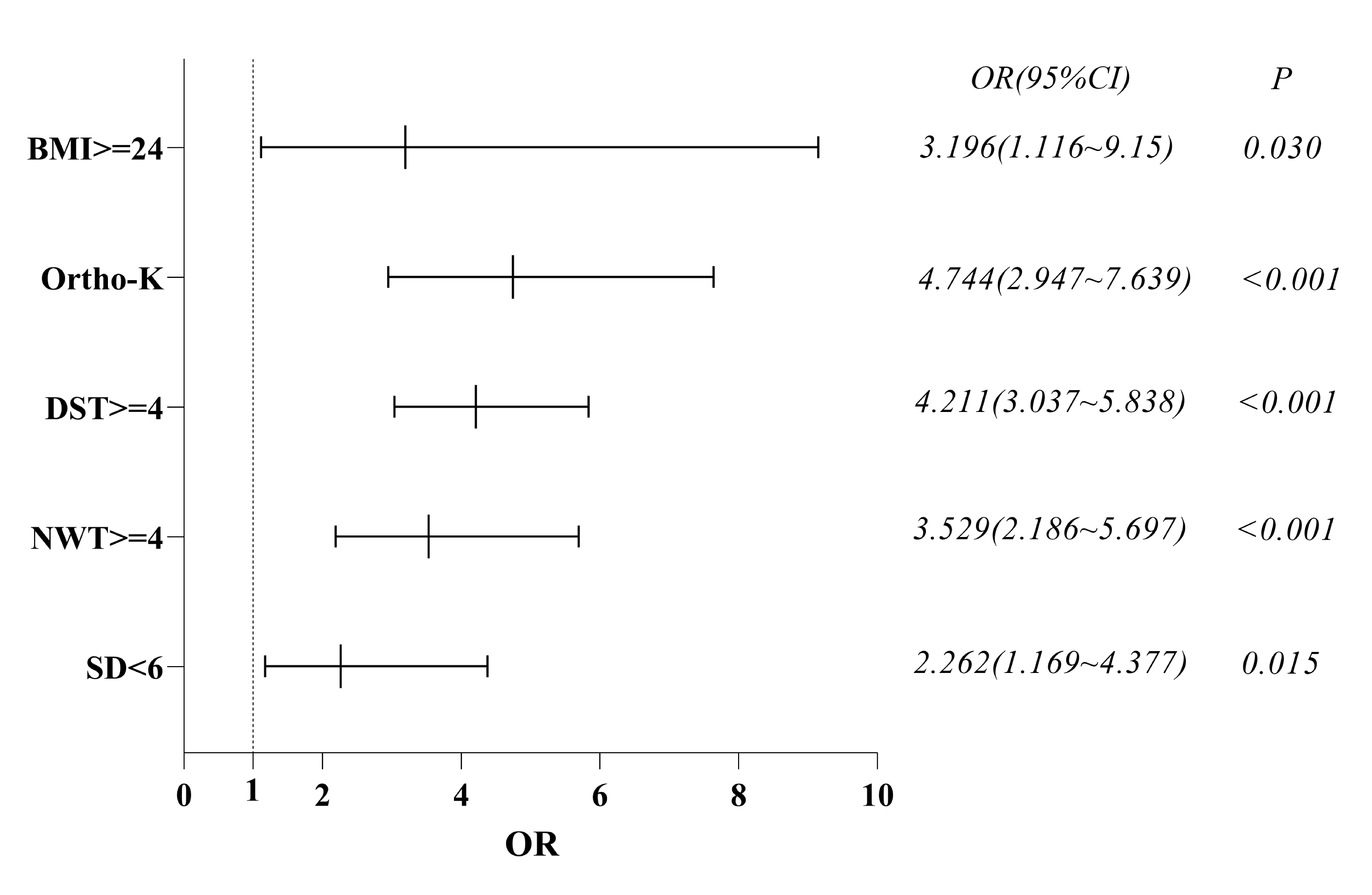

Supplement: Supplementary Figure 2 — Forest plot of independent risk factors for objective dry eye in myopic children. [file Image_2.tif]

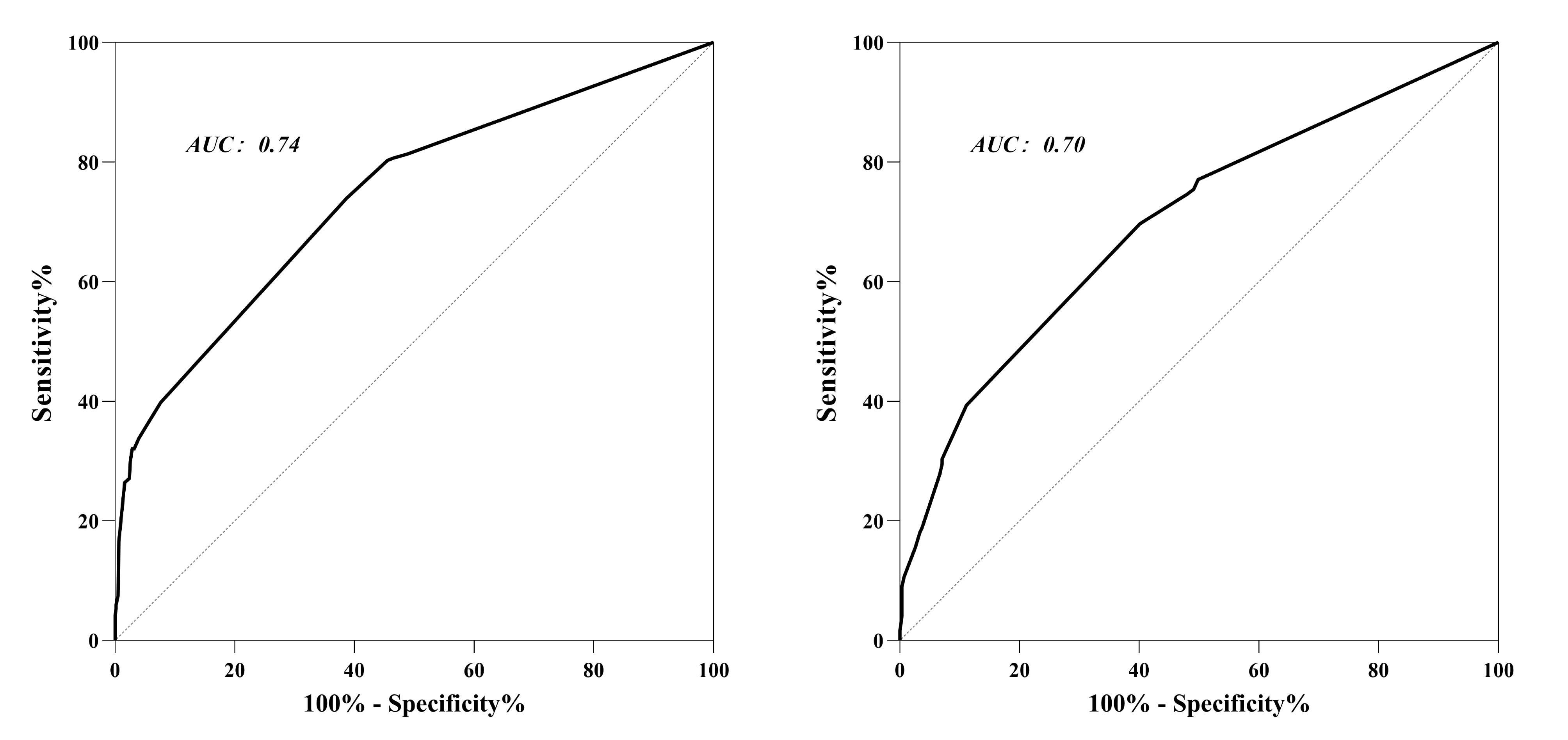

Supplement: Supplementary Figure 3 — ROC curves for model discrimination: (left) training set, (right) validation set. [file Image_3.tif]

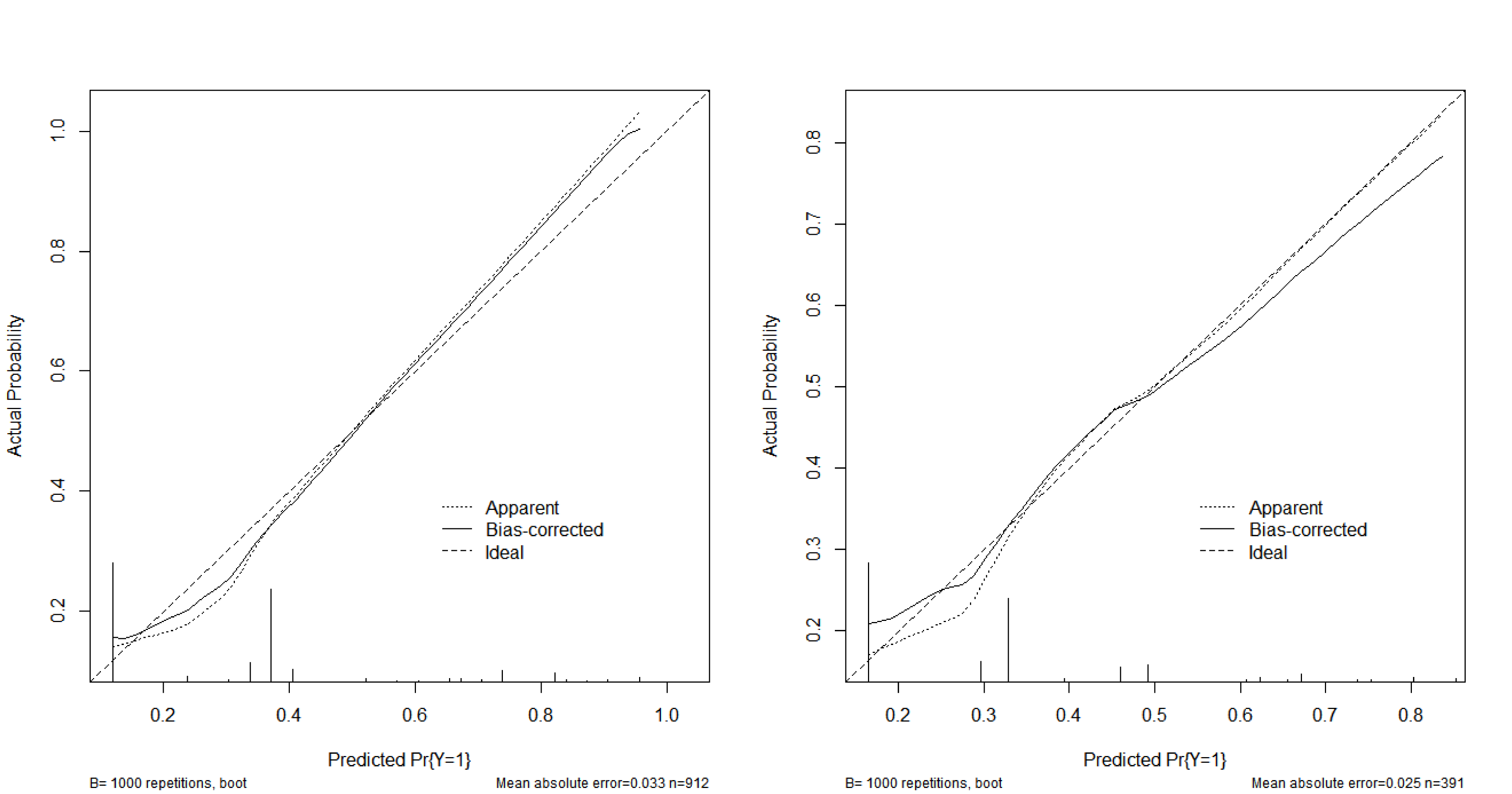

Supplement: Supplementary Figure 4 — Calibration plots for nomogram in (left) training set and (right) validation set. [file Image_4.tif]

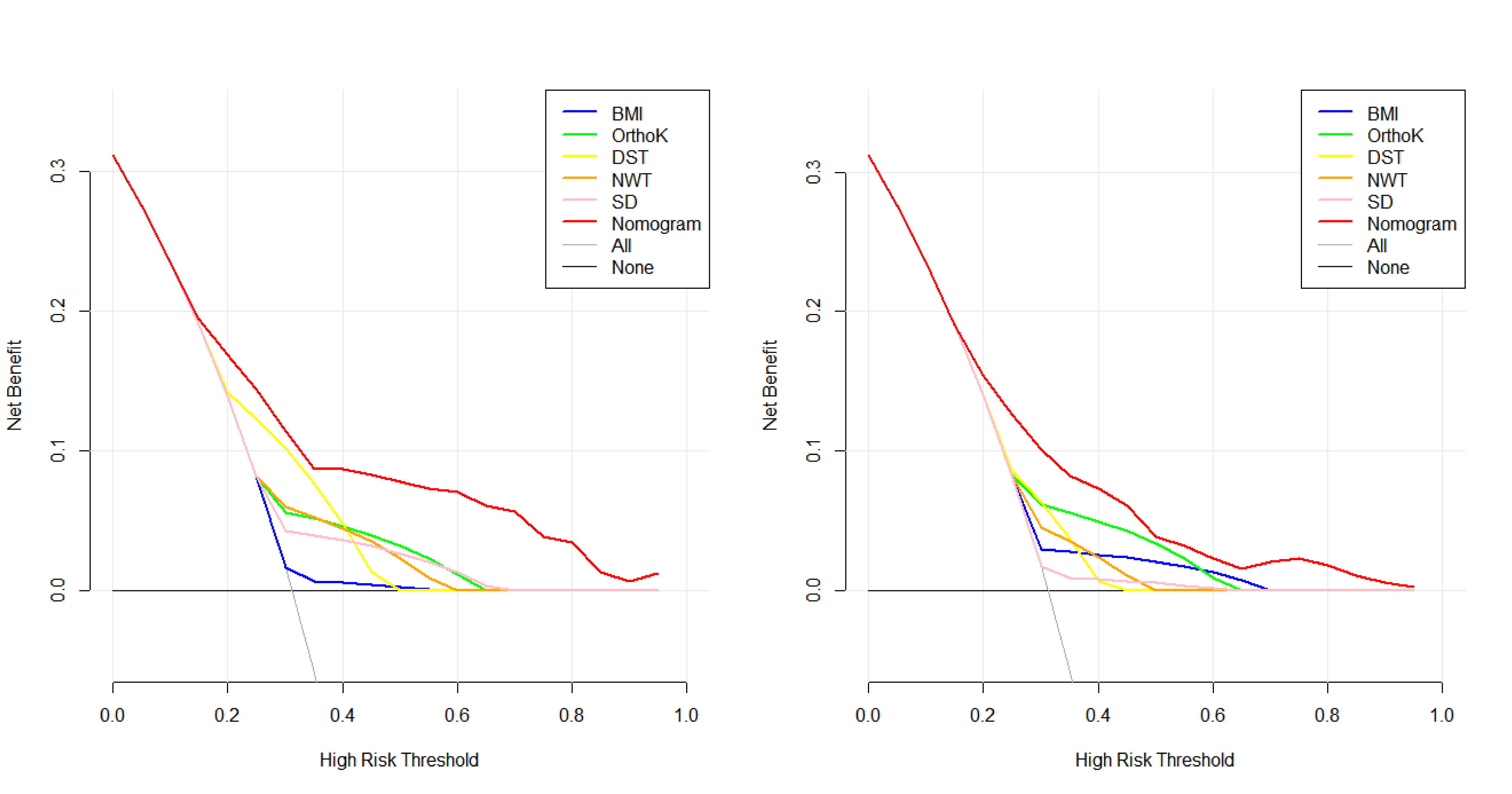

Supplement: Supplementary Figure 5 — Decision curve analysis (DCA) comparing net clinical benefit of the nomogram and individual factors in the (left) training and (right) validation sets. [file Image_5.tif]
